# From multiple measles genotype D8 introductions in 2024 to sustained B3 local transmission in and around Milan, northern Italy, January to April 2025

**DOI:** 10.2807/1560-7917.ES.2025.30.20.2500315

**Published:** 2025-05-22

**Authors:** Clara Fappani, Maria Gori, Silvia Bianchi, Lucia Tieghi, Daniela Colzani, Sabrina Senatore, Marino Faccini, Priscilla Pasutto, Luca Imeri, Luigi Vezzosi, Gabriele Del Castillo, Simone Villa, Danilo Cereda, Silvia Gioacchini, Paola Bucci, Raoul Fioravanti, Emilio D’Ugo, Melissa Baggieri, Fabio Magurano, Antonella Amendola

**Affiliations:** 1 Department of Health Sciences, Università degli Studi di Milano, Milan, Italy; 2 Coordinated Research Centre “EpiSoMI”, Università degli Studi di Milano, Milan, Italy; 3 Health Protection Agency of the Metropolitan Area of Milan, Milan, Italy; 4 Directorate General for Health of the Lombardy Region, Milan, Italy; 5 Department of Infectious Diseases, Istituto Superiore di Sanità, Rome, Italy

**Keywords:** measles surveillance, molecular epidemiology, outbreak, endemicity, vaccination coverage, vaccine preventable diseases

## Abstract

An outbreak of measles virus genotype B3 is ongoing in Milan and surrounding areas since February 2025, with 27 cases identified in 32 laboratory-confirmed measles cases. Most cases were locally acquired and young adults. Phylogenetic analysis indicated the presence of a unique lineage closely related to strains circulating in Morocco. The lack of epidemiological links between several affected individuals suggests case numbers are being underestimated. The continuous transmission raises concerns about the potential re-establishment of endemic circulation in northern Italy.

In 2024, the Metropolitan City of Milan and surrounding areas in northern Italy (ca 4 million people) experienced a measles epidemic caused by multiple introductions of different genotype D8 lineages, which led to small outbreaks, mostly linked to importation events and/or vulnerable population groups, without evidence of sustained local circulation [1]. As Subnational Reference Laboratory of the Italian measles and rubella surveillance network (MoRoNet), we now report a new surge of measles in the same area in 2025, driven by a unique lineage of genotype B3, causing locally acquired cases.

## Measles surveillance activities in 2025

From January to April 2025, we investigated 42 suspected measles cases in the Metropolitan City of Milan and surrounding areas. Demographic and epidemiological data were collected through a standardised report form, epidemiological investigations were conducted by local public health authorities, and case definitions were applied according to the World Health Organization (WHO) guidelines [2]. Paired oropharyngeal swabs and urine samples were analysed by real-time RT-PCR [3], confirming measles infection in 32 cases. No differences between sexes were observed (18 males, 14 females), and the median age was 36.5 years (range: 1–48 years). Twelve cases were hospitalised, two with severe pneumonia. One case was in their last trimester of pregnancy. Two occupational cases likely contracted the infection in a healthcare setting. Seven of the 32 cases were vaccinated, with one individual vaccinated with two doses of measles-containing vaccine. According to the epidemiological investigation, most (23/32) cases were reported as locally acquired (indigenous).

**Figure 1:**
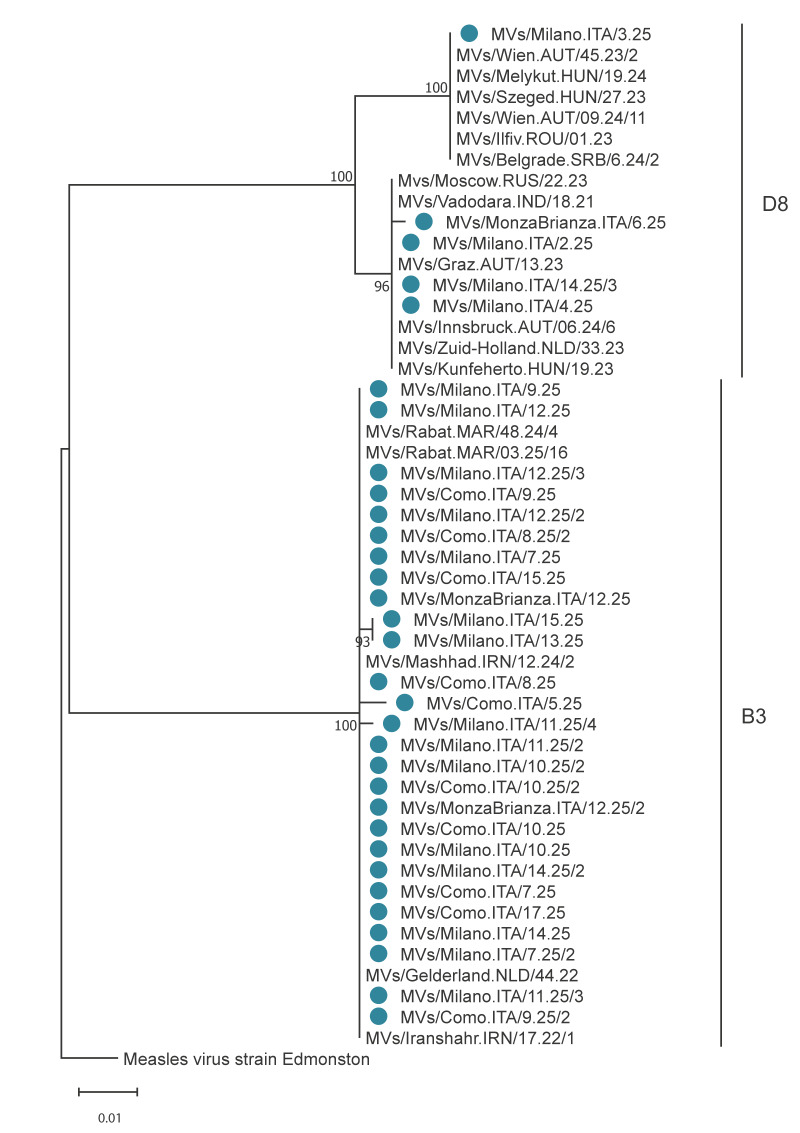
Phylogenetic analysis of measles strains identified in the Metropolitan city of Milan and surrounding areas, Italy, January 2025–April 2025 (n = 32)

Measles viruses (MeV) from all confirmed cases were successfully genotyped by amplifying and sequencing the highly variable C-terminal region of the nucleoprotein coding gene (N-450) [4]. All sequences were deposited in GenBank (https://www.ncbi.nlm.nih.gov/genbank) [5] and the WHO Measles Virus Nucleotide Surveillance (MeaNS2) database [6]. Identified strains were classified as genotype D8 (n = 5) and B3 (n = 27) (Figure 1). Genotype D8 strains showed identity between 97.6% and 100%, and clustered with sequences identified between 2021 and 2024 in Austria, Hungary, India, the Netherlands, Serbia, Romania and Russia. Genotype B3 strains showed identity between 99.3% and 100%, and clustered with sequences identified between 2022 and 2025 in Iran, Morocco and the Netherlands.

The evolutionary history was inferred by using the maximum likelihood method and Kimura 2-parameter model of nucleotide substitutions, and the tree with the highest log-likelihood (−925.20) is shown. Branch support (1,000 bootstrap iterations) [18] is provided next to nodes. The initial tree for the heuristic search was selected by choosing the tree with the superior log-likelihood between a neighbour-joining (NJ) tree [19] and a maximum parsimony (MP) tree. The NJ tree was generated using a matrix of pairwise distances computed using the p distance. The MP tree had the shortest length among 10 MP tree searches, each performed with a randomly generated starting tree. The analytical procedure encompassed 46 coding nucleotide sequences using first, second, third and non-coding positions with 450 positions in the final dataset. Evolutionary analyses were conducted in MEGA12 [20] using up to seven parallel computing threads. The tree was constructed using genotype A strain Edmonston as the outgroup. Blue circles identify measles strains detected in the area under surveillance.

The five cases with D8 were detected between calendar weeks 2 and 14 2025. None of these cases was acquired locally: four had visited another Italian region or Romania and for one case, the source of infection was unknown. Overall, three distinct sequence identifiers (DSIds) were identified (5963, 8350 and 8996).

## Ongoing circulation of a B3 lineage in Milan and surrounding areas

**Figure 2:**
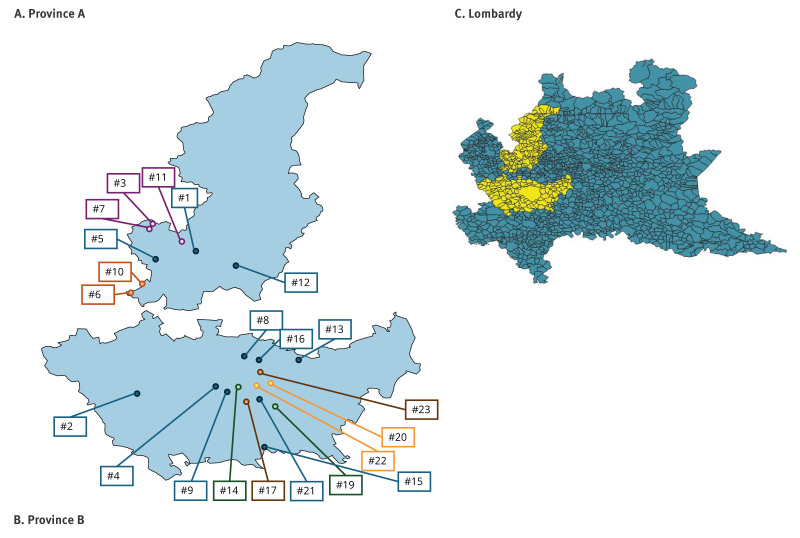
Geographical distribution of genotype B3 measles cases in Province A and Province B, northern Italy, January–April 2025 (n = 27)

The B3 genotype was introduced into the area under surveillance in week 5 2025 in Province A by a case returning from travel (Case #1) (Table, Figure 2). In week 7 2025, we detected a locally acquired B3 case in the same area (Case #3), with no known direct link with Case #1 or other persons who experienced rash in the previous 7–21 days. From week 7 to week 17 2025, we identified eight more measles cases in the same province. Overall, three small outbreaks involving seven cases were observed (Table, Figure 2). The other four cases were reported as sporadic.

**Table:** Confirmed genotype B3 measles cases in the Metropolitan City of Milan and surrounding areas, Italy, January–April 2025 (n = 27)

ID	Province	Month	Rash onset (week)	Vaccination status	Doses (n)	Hospitalisation	Source	MeaNS2 DSId	Sequence name
#1	A	February	5/2025	No	NA	Yes	Travel	8495	MVs/Como.ITA/5.25
#2	B	February	7/2025	Post exposure vaccine		No	Travel^a^	6418	MVs/Milano.ITA/7.25
#3	A	February	7/2025	No		Yes	Indigenous	6418	MVs/Como.ITA/7.25
#4	B	February	7/2025	Unknown		Yes	Travel	6418	MVs/Milano.ITA/7.25/2
#5	A	February	8/2025	No		No	Indigenous	6418	MVs/Como.ITA/8.25
#6	A	February	8/2025	No		No	Indigenous	6418	MVs/Como.ITA/8.25/2
#7	A	February	9/2025	Post exposure vaccine		Yes	Family (linked to Case #3)	6418	MVs/Como.ITA/9.25
#8	B	February	9/2025	No		Yes	Indigenous	6418	MVs/Milano.ITA/9.25
#9	B	March	10/2025	Yes	Unknown	Yes	Indigenous	6418	MVs/Milano.ITA/10.25
#10	A	March	10/2025	No	NA	No	Family (linked to Case #6)	6418	MVs/Como.ITA/9.25/2
#11	A	March	10/2025	No		No	Hospital (linked to Case #3)	6418	MVs/Como.ITA/10.25
#12	A	March	10/2025	No		Yes	Indigenous	6418	MVs/Como.ITA/10.25/2
#13	B	March	10/2025	Unknown		Yes	Indigenous	6418	MVs/Milano.ITA/10.25/2
#14	B	March	11/2025	No		Yes	Indigenous	6418	MVs/Milano.ITA/11.25/2
#15	B	March	11/2025	No		No	Indigenous	6418	MVs/Milano.ITA/11.25/3
#16	B	March	11/2025	Yes	2	No	Hospital^a^	8339	MVs/Milano.ITA/11.25/4
#17	B	March	12/2025	Unknown	NA	No	Indigenous	6418	MVs/Milano.ITA/12.25
#18	C	March	12/2025	No		No	Work-related^a^	6418	MVs/MonzaBrianza.ITA/12.25
#19	B	March	12/2025	No		NA	Work-related (linked to Case #14)	6418	MVs/Milano.ITA/12.25/2
#20	B	March	12/2025	No		No	Indigenous	6418	MVs/Milano.ITA/12.25/3
#21	C	April	12/2025	Yes	1	No	Indigenous	6418	MVs/MonzaBrianza.ITA/12.25/2
#22	B	March	13/2025	No	NA	No	Travel	6418	MVs/Milano.ITA/13.25
#23	B	April	14/2025	No		NA	Family (linked to Case #20)	6418	MVs/Milano.ITA/14.25
#24	B	April	14/2025	Yes	1	No	Family (linked to Case #17)	6418	MVs/Milano.ITA/14.25/2
#25	A	April	15/2025	Yes	1	No	Indigenous	6418	MVs/Como.ITA/15.25
#26	B	April	15/2025	No	NA	No	Travel	9418	MVs/Milano.ITA/15.25
#27	A	April	17/2025	Yes	1	No	Indigenous (linked to Case #25)	6418	MVs/Como.ITA/17.25

DSId: distinct sequence identifier; ID: identification code; Indigenous: locally acquired; MeaNS2: World Health Organization Measles Virus Nucleotide Surveillance; NA: not applicable.

^a^ This case was a contact of a laboratory-confirmed measles case residing outside the area under surveillance.

A colour pattern different from blue identifies a direct linkage between cases.

Starting from week 7 2025, we detected the same B3 lineage in the neighbouring Province B (south of province A) in a case linked to a traveller (Case #2) and in a case with a history of travel (Case #4). Since week 9 2025, in the same province, we observed the spread of the B3 lineage among 11 locally acquired cases. We identified five small outbreaks (Table, Figure 2). Two additional imported cases (Cases #22 and #26) were detected in weeks 13 and 15 2025, in travellers. In week 12 2025, we confirmed two cases in the neighbouring Province C (south of province A and north of province B, Cases #18 and #21).

By week 18 2025, four distinct sequence identifiers (DSIds) (6418, 8495, 8339 and 9418) were assigned, with 6418 the most prevalent. Four confirmed cases (#3, #4, #5 and #8) had attended mass gathering events in Province B in the 7–21 days before rash onset; of these, two lived in Province A and had no direct link with cases in Province B. Of the travel-associated cases, the countries involved were Egypt, France, Morocco and Spain.

## Comparison with the 2024 measles epidemic activity

Although the number of fever and rash cases observed in the same months (January–April) in the area under surveillance in 2024 and 2025 were fairly similar (36 cases in 2024 vs 42 cases in 2025), the laboratory-confirmed measles cases increased in 2025 nearly twofold compared with 2024 (32/42 in 2025 vs 14/36 in 2024; Fisher’s exact test (one-tailed); p value = 0.0009). Furthermore, while in 2024, 9 of 14 confirmed cases were travel-related, in 2025, most of the cases were reported as locally acquired (imported cases: 9/14 in 2025 vs 9/32 in 2024; p value = 0.02) [1]. Lastly, the measles epidemiology in the first 4 months of 2024 was characterised by the co-circulation of three D8 lineages, while in 2025, measles epidemic activity was mainly sustained by a unique B3 lineage.

## Discussion

According to the most recent update by the European Centre for Disease Prevention and Control (ECDC), Italy is among the five countries with the highest number of reported measles cases in the European Union/European Economic Area (EU/EEA) countries [7]. In the WHO European Region, in 2024, the number of measles cases reached the highest level since 1997 [8]. Italy was among the most affected countries with 1,097 cases [9] and lost its near-elimination status (< 10 cases per million population), with an incidence rate of 17.7 cases per million population [9-11].

Overall, the scenario of 2024 was characterised by the co-circulation of five D8 and two B3 lineages with different importation sources (unpublished data). Currently, the pattern of circulating MeV strains is characterised by the spread of a unique lineage of genotype B3, presumably representing a single chain of transmission [12], mostly sustained by locally acquired cases. This scenario is typical of countries experiencing reintroduction of measles, with the occurrence of outbreaks associated with a single genotype with nearly identical sequences [12]. The integration of thorough epidemiological investigation with molecular characterisation of outbreak strains suggested both multiple introductions in 2024 and uninterrupted transmission of a single B3 lineage in 2025 in and around the Metropolitan City of Milan.

Most cases were geographically and temporally clustered, with dates of rash onset occurring 7–21 days apart, suggesting the presence of an epidemiological pathway [2]. A measles lineage that propagated continuously for a prolonged period in a given area raises concerns about the potential re-establishment of endemic transmission.

In line with national data [10], the ongoing outbreak is affecting mainly unvaccinated young adults and adults. The high vaccination coverage in Lombardy, the region surrounding Milan, currently 96.06% and 93.63% for the first and second dose, respectively, has successfully limited transmission in younger age groups. The cases described here are within pockets of susceptible individuals, who were not exposed to natural infection due to the absence of considerable epidemic circulation and did not receive the vaccine either because immunisation was not yet mandatory or was not widely offered during their childhood. It is therefore crucial to implement immunisation catch-up programmes, as recommended by local and national authorities (Regional regulation number G1.2024.0004194, 6 February 2024) [10].

Almost all the N-450 sequences of B3 measles strains identified in the area under surveillance in 2025 are identical to a B3 strain circulating in Morocco in 2024–2025, where a large measles outbreak is ongoing [13,14]. French public health authorities have recently reported a notable increase in measles cases with a history of travel to Morocco since the start of the year [15]. We identified only two returning travellers from Morocco and one from France, suggesting that the circulating B3 lineage became established in Milan and surrounding areas, causing cases locally.

The genetic characterisation of the circulating MeVs, integrated with case-based epidemiological investigation, provides valuable insight into transmission dynamics. In our context, this led to the identification of the introduction of a B3 lineage that spread in and around Milan, giving rise to an ongoing episode of uninterrupted transmission from a genetic standpoint. Although we have hypothesised a single chain of measles transmission, we cannot exclude the possibility that some cases with a history of travel may have gone undetected by the surveillance system, resulting in missing links between the identified cases.

Endemic transmission is defined by the WHO as a chain of transmission that circulates continuously in a given area for more than 12 months [16]. While we have not yet reached that threshold, the current pattern is worrisome and warrants remaining vigilant to avoid the potential re-establishment of endemic transmission. As recently highlighted by the ECDC, considering the seasonality of measles, cases may continue to increase in the coming months [17].

## Conclusion

The Metropolitan City of Milan and the surrounding areas are experiencing a surge in the number of measles cases driven by a single genotype B3 lineage, primarily affecting unvaccinated adults. Our findings raise concerns about the potential return of measles as an endemic disease, underscoring the urgent need to implement all necessary countermeasures (i.e. close immunity gaps, maintain high-quality surveillance) to prevent this scenario from becoming a reality.

## Data Availability

Sequence data associated with this study have been deposited in the MeaNS2 database (https://who-gmrln.org/means2) and in GenBank (https://www.ncbi.nlm.nih.gov/genbank). The accession numbers are pending and will be updated here once available.
