# Addendum for Euro Surveill. 2025;30(21)

**DOI:** 10.2807/1560-7917.ES.2025.30.21.250529A

**Published:** 2025-05-29

**Authors:** Eurosurveillance editorial team

**Affiliations:** 1 European Centre for Disease Prevention and Control (ECDC), Stockholm, Sweden

In the article ‘From multiple measles genotype D8 introductions in 2024 to sustained B3 local transmission in and around Milan, northern Italy, January to April 2025’ by Fappani et al. published in *Eurosurveillance* on 22 May 2025, the GenBank accession numbers of the genomic sequences obtained in the study were added to the Data availability statement on 26 May 2025.

